# Adaptive Trust Evaluation Model Based on Entropy Weight Method for Sensing Terminal Process

**DOI:** 10.3390/e27020200

**Published:** 2025-02-14

**Authors:** Tao Li, Yanyi Zhang

**Affiliations:** 1 School of Cyber Science and Engineering, Southeast University, Nanjing 211189, China; lit@seu.edu.cn; 2 Purple Mountain Laboratories, Nanjing 211111, China; 3 Frontiers Science Center for Mobile Information Communication and Security, Southeast University, Nanjing 211189, China

**Keywords:** process security, trust evaluation, trust value, auto-correlation, k-means cluster analysis, entropy weight method

## Abstract

Remote sensing (RS) has been widely used for data acquisition, monitoring, control, and intelligent decision-making. However, most of them are unattended and easily become the target of attack, which means there are still some risks in the sensing terminal processes. Therefore, trust evaluation of the processes associated with the sensing terminal is necessary. The existing trust evaluation model based on the sensing terminal process has some defects, such as low performance, low precision, and difficulty in effectively identifying malicious processes in the sensing terminal. In this paper, an adaptive trust evaluation model based on the entropy weight method is proposed to detect the sensing terminal process (PB-ATEM). By establishing two kinds of trust values, the direct trust value and the reciprocal trust value, we can comprehensively judge whether the process is trustworthy. For the direct trust value, we can dynamically capture the auto-correlation value of processes and establish a dynamic reward and punishment function to improve the response to malicious processes. For reciprocal trust values, k-means cluster analysis is used to classify processes, and the optimal entropy weight method is proposed to calculate the direct trust weight and reciprocal trust weight of each process more accurately, which accelerates the exposure of malicious processes. Finally, these two trust values are weighted to obtain the integrated trust value. The simulation results show that PB-ATEM can respond quickly to malicious processes. Compared with the existing trust evaluation models, it has higher detection accuracy and better ability to identify malicious processes.

## 1. Introduction

With the development of sensor technology, including the rapid development and maturity of micro-electromechanical systems (MEMSs) and related interfaces and signal processing technologies, micro-sensor networks with sensing, computing, and communication capabilities have attracted great attention. The development of these technologies provides the basis for the application of remote sensing technology. In sensing terminal devices, processes (such as device management process, data collection process, and communication process) are responsible for performing various tasks such as data acquisition, signal processing, communication, and system control. However, due to limited computing power and weak security measures, these processes are easy targets for attacks. Hence, the security problems of sensing terminal processes are increasingly appearing in the remote sensing field. Actually, the sensing terminals are generally in an unattended state. If some of the key processes responsible for sensing and sending data in the sensor are illegally invaded and then the data are leaked, it will cause incalculable losses. Therefore, process security in the sensing terminal has become a hot spot in recent years. Process security refers to the protection of the sensor’s system processes from accidental or malicious conditions and aims to ensure the continuous and reliable operation of the system. Process security can also ensure that information services are not interrupted, allowing for business continuity [1] in the sensing terminal.

With the continuous iteration and update of software, process security in the sensing terminal has received much attention in the field of information security research. Attack methods also tend to be diversified. Especially in recent years, with the continuous improvement of hacker technology, the processes in the sensing terminal face various security risks, such as tampering, permission promotion, process hijacking, and denial-of-service attacks, which bring great security risks to user management. To prevent the illegal operation of malicious processes and unnecessary programs, it is particularly important to analyze the behavior of the process [2].

Trust evaluation is an effective way to measure whether a process is trustworthy or not. It uses mathematical methods to establish a trust model and evaluate various process parameters, and the results are highly persuasive. Therefore, it is important to establish a reasonable trust model to evaluate process security. In recent years, the object-oriented trust model has become a focus of trust evaluation research, mainly focusing on cloud computing security, Internet of Things (loT) nodes communication security, and process behavior analysis. At present, the research on the object-oriented trust model is mainly divided into two directions: strategy-based trust mechanism and trustworthiness-based trust mechanism. The strategy-based trust mechanism manages certificates through public key infrastructure (PKI) [3] and requires strict authority to maintain and demonstrate a trust relationship. The trustworthiness-based trust mechanism [4] detects the object with a low trust value and deals with it by studying the context change and the trust relationship with other objects.

For the above two trust models, a strategy-based trust mechanism is usually applied in the cryptography system. The trust model requires not only trusted authority but also complex cryptographic computation, which requires high requirements for the selection of trust algorithms and makes it difficult to realize a dynamic detection. In addition, due to the resource limitation of sensor devices, such as computing, communication bandwidth, and processing, a lightweight and real-time model must be applied. Therefore, this paper uses the trustworthiness-based trust mechanism, which can not only achieve the lightweight of the model but also dynamically track the change in the processes. However, in existing studies on process behavior analysis using a trustworthiness-based trust mechanism, the accuracy of the trust models proposed by these studies is generally low, and adaptability is poor. The existing trust evaluation models for sensing terminal process should have the following characteristics: lightweight, efficient, real-time, adaptive, and multi-dimensional behavior analysis.

To solve these problems, this paper proposes an adaptive trust evaluation model based on the entropy weight method for the sensing terminal process (PB-ATEM). This paper makes the following contributions:

- We select some common performance parameters of each process, such as CPU usage, memory usage, I/O, etc., to calculate the unique process identification and the auto-correlation value of process context changes.
- We set up a dynamic trust model to capture the change in the process context in real time. A dynamic reward and punishment function is set up to accelerate response speed to malicious processes and enhance the identification ability of malicious processes.
- We use k-means cluster analysis to classify processes according to the direct trust value and auto-correlation value. K-means clustering has the characteristics of simplicity, high accuracy, and fast clustering, so malicious processes can be effectively distinguished from normal processes.
- Based on the results of the k-means cluster analysis, we propose an optimal entropy weight method to determine the adaptive weight. The entropy weight method is suitable for the situation where the correlation between indicators is weak and no single parameter can directly determine the evaluation result (such as CPU usage and memory usage are not directly related) and can better deal with the interaction between indicators.

The first section of this paper introduces the relevant background of process security in the sensing terminal, points out the existing problems in the research content of process behavior analysis, and presents the improvements to solve these problems. In the second section, we introduce the relevant research on process security behavior analysis and security trust models, put forward some shortcomings of these models, and explain the improvement plan. In the third section, according to the improvement scheme proposed in the second section, an adaptive trust evaluation model based on the entropy weight method is proposed to detect the untrusted sensing terminal process. This model captures process performance parameters in real time, calculates auto-correlation value according to the changes in context, and obtains direct trust values and reciprocal trust values according to the auto-correlation value. Finally, the two trust values are weighted by adaptive weights to obtain the integrated trust value. In the fourth section, this paper chooses two representative trust evaluation models to compare with PB-ATEM. Based on the analysis, PB-ATEM has a faster response speed and higher detection accuracy. In the fifth section, the research content of this paper is summarized, and future works are proposed.

## 2. Related Works

Currently, research in the field of process security is divided mainly into two directions: process security behavior analysis and the process security trust evaluation model. In terms of security behavior analysis, Li et al. [5] proposed the corresponding security measures for sensing terminals of IoT systems, such as physical security, access security, communication security, equipment security, and data security. Furthermore, they have verified it with experiments. Arabo et al. [6] analyzed the behavior and performance parameters of processes and used machine learning to identify malicious processes. Steingartner et al. [7] designed a hybrid threat conceptual model of a new deception method based on the process security defense mechanism to reveal vulnerabilities in process security. Pedro et al. [8] studied the latest developments in device behavior in terms of application scenarios, behavior sources, processing, and evaluation techniques for device security. Jabar et al. [9] discussed different methods used in network security and aim to defend against Advanced Persistent Threat (APT) attacks, which is one kind of device attack behavior. Agrawal, Alka et al. [10] used a fuzzy-based symmetrical decision-making approach to assess the security of software with respect to tactics. The Fuzzy Analytic Network Process (F-ANP) is applied to evaluate the weights of criteria, and the fuzzy-symmetrical technique for Order of Preference by Similarity to Ideal Solution (TOPSIS) is used to determine the impact of alternatives. Mittal, Himani [11] stated that several types of security threats that have existed since the time networking has evolved include malware, Trojans, viruses, and denial-of-service attacks. They also reviewed several measures to address these threats, including logging, anti-malware, network security methods, and encryption methods.

In terms of security trust evaluation model research, Chen et al. [12] proposed a trust evaluation model, NPTM, based on user behavior data in cloud computing, which evaluates the user’s trustworthiness according to the integrated trust value obtained by the integrated weighting of the user’s historical behavior, reciprocal behavior, and direct behavior. Aiming at the security and reliability of the cloud service system, Gong et al. [13] proposed a new malicious rating punishment method based on three criteria: cost, profit, and risk. Wang et al. [14] proposed a dynamic cloud service trust evaluation model based on Service Level Agreement (SLA) and privacy awareness in order to solve the cloud service allocation problem of applications. Muchahari et al. [15] proposed a cloud management architecture composed of a cloud service registry and discovery, which serves as cloud providers’ registry and lists their respective trust values. It includes a Trust Calculator that calculates cloud service providers’ (CSPs) trust based on feedback from two parameters, namely Service Level Agreement (SLA) and Quality of Service(QoS). Mohammed et al. [16] proposed a trust evaluation model to determine the degree of trust of cloud consumers, using Particle Swarm Optimization (PSO) technology to evaluate consumers. Rieke et al. [17] proposed a predictive security evaluation method for the security of electronic money transactions and applied a Predictive Security Analyzer (PSA) to identify risk problems of electronic money transactions. Cui et al. [18] proposed a user behavior trust evaluation model based on the improved Analytic Hierarchy Process (AHP), which sets game theory for security in cyberspace.

The above research models generally identify malicious processes through trustworthiness-based evaluation methods, providing a theoretical basis for the research content of this paper. However, existing research still has the following problems to be solved:

- The performance parameters selected in the trust model are only the performance parameters at the current state; however, the context of performance parameters changes before and after the exiting state are not considered. The limitation of parameters makes it impossible to fully reflect the process’s changes.
- When calculating trust value, it ignores some non-malicious factors, such as the system’s own environmental fluctuations, power consumption, and so on, which will lead to misjudgment.
- The self-adaptability is not clear enough. The monitoring of the process should be a dynamic process, its performance parameters should be captured in real time, and the trust value and trust weight should be dynamically updated.
- The weight assignment of trust values is relatively simple, and there is no effective method to respond quickly to the intrusion of malicious processes.

To solve the above problems, an adaptive trust evaluation model PB-ATEM is proposed. Our model calculates the unique process identification of each process and the auto-correlation value of the process identification changes. Additionally, we set a dynamic reward and punishment function to accelerate the identification of malicious processes. And the k-means cluster analysis and the optimal entropy weight method are used to distinguish malicious processes from normal processes to reduce the model’s misjudgment of normal processes.

## 3. Adaptive Trust Evaluation Model Based on Process Behavior Analysis

**Figure 1:**
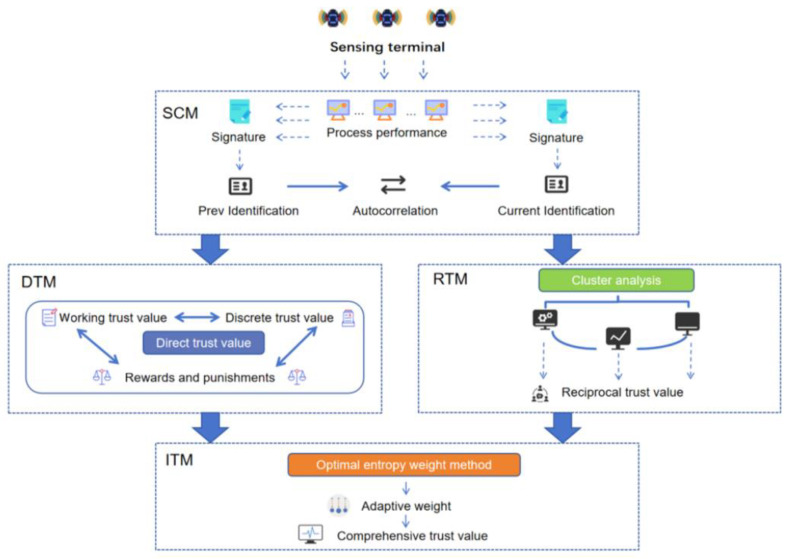
PB-ATEM Framework.

In order to complete the improvements above, we divided PB-ATEM into four modules according to different parameter categories: signature computing module, direct trust module, reciprocal trust module, and integrated trust module. Figure 1 shows the framework of the model. The process of each module is as follows:

Signature computing module (SCM): According to the CPU usage, memory usage, and I/O of each process, the unique hash signature of the current time is generated, recorded as the unique identification of the current time of the process. The auto-correlation value of its context change is calculated according to the unique identification, as the basis for judging the changes in processes.

Direct trust module (DTM): Auto-correlation value generated by the signature computing module of the process context in the time series is used to determine the direct trust value. To respond to the change in the process more effectively, the reward and punishment function is designed.

Reciprocal trust module (RTM): The K-means cluster analysis is used to classify processes according to trust values and auto-correlation values. Then, the discrete weight is calculated according to the discretization degree of the process in the cluster, and the reciprocal trust value is determined according to the direct trust value and discrete weight.

Integrated trust module (ITM): An optimal entropy weight method is proposed to calculate the adaptive weights. Firstly, the parameter matrix of all processes in each moment is constructed. According to the classification results of cluster analysis, the evaluation weights of different types of processes are calculated by optimizing the entropy weight method. From the evaluation weights, direct trust weights and reciprocal trust weights are obtained. Finally, the integrated trust value is obtained according to the direct trust value and reciprocal trust value.

**Figure 2:**
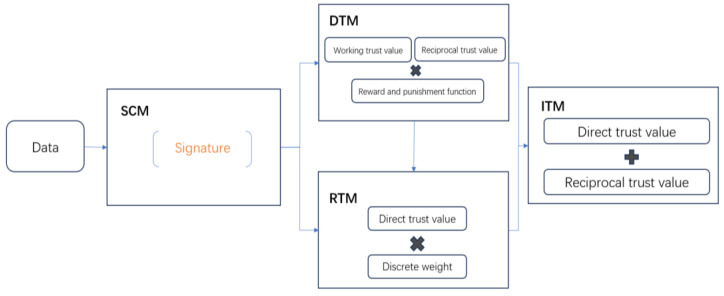
PB-ATEM working flow.

The workflow and correlation of the above four modules of PB-ATEM are shown in Figure 2. First, the auto-correlation values of processes obtained from SCM are used to calculate the working trust values and discrete trust values in DTM, and the direct trust values are obtained by introducing the reward and punishment function into DTM. In RTM, the discrete weight of each process is obtained through an analysis of k-means, and the reciprocal trust values are calculated according to the direct trust values calculated by the DTM. Finally, the integrated trust values of the process are obtained by weighting the direct trust values and the reciprocal trust values.

Based on the overview of PB-ATEM, the following Section 3.1, Section 3.2, Section 3.3 and Section 3.4 mainly introduce the operation mechanism of the four constituent modules of PB-ATEM.

### 3.1. Signature Computing

In the existing attack mode, most malicious processes must expose exceptions in some performance parameters when they behave maliciously. No matter how malicious programs are hidden, they must reveal flaws in some performance parameters. Each process must be distinguished one by one in a certain way to effectively identify malicious processes. We used a signature method to generate a unique identification number for each process and extracted different performance parameters of the process, including CPU usage, memory usage, and I/O. These three parameters are recorded as α, β, and γ. The Performance Index Sequence (*PIS_i_*) of a process is as follows:

(1) $$PIS_{i}=[\vec{\alpha_{i}},\;\vec{\beta_{i}},\;\vec{\gamma_{i}}]$$

where *PIS_i_* indicates the performance index sequence of the *i*-th process, $\vec{\alpha_{i}}$ represents the average CPU usage of the process, $\vec{\beta_{i}}$ represents the average memory usage, $\vec{\gamma_{i}}$ represents the average I/O.

After parameter collection is completed, the hash signature values of each parameter are calculated and the unique identification *U_i_* (*t*) of each process at time t is generated, which is expressed as follows:

(2) $$U_{i}\;(t)=[Sig\;(\vec{\alpha_{i}}),\;Sig\;(\vec{\beta_{i}}),\;Sig\;(\vec{\gamma_{i}})]$$

where *U_i_* (*t*) indicates the unique signature identifier of the *i*-th process at the *t*-th time. Sig ($\vec{\alpha_{i}}$), Sig ($\vec{\beta_{i}}$), Sig ($\vec{\gamma_{i}}$), respectively, indicate the signature of the three parameters.

After the unique identification of the process is obtained, the above changes in the process need to be analyzed. The auto-correlation value [19] *C_i_* (*t*) is used to represent the context changes in the process, which is expressed as follows:

(3) $$C_{i}\;(t)=\;\frac{\sum_{t=k+1}^{N}\left(U_{i}(t)-\mu \right)\cdot \left(U_{i}(t-k)-\mu \right)}{\sum_{t=1}^{N}\left(U_{i}(t)-\mu \right)\cdot \left(U_{i}(t)-\mu \right)}$$

where *C_i_* (*t*) represents the auto-correlation value of the process identification change in the *i*-th process at the *t*-th time point and the previous *k* time points, *N* represents the length of the time series, *k* represents the timestamp. Where *C_i_* (*t*) represents the auto-correlation value of the process identification change in the *i*-th process at the *t*-th time point and the previous *k* time points, *N* represents the length of the time series, *k* represents the timestamp, μ is the mean of the process identification in the time series, for the convenience of the following calculation, the auto-correlation values are represented by *C*(*t*).

### 3.2. Direct Trust Value Calculation

The module dynamically updates its direct trust values on the changes in auto-correlation values to achieve the purpose of real-time monitoring of the process. First, when the process starts to run, its auto-correlation value is usually high or low, and the auto-correlation value becomes stable after a short period of time without other intervention. The maximum auto-correlation value after stability is denoted as $C_{max}$. And the empirical value is defined as μ. The working trust value of the i-th process at *t*-th time can be expressed as:

(4) $$W_{i}(t)=\frac{C(t)\;+\mu }{C_{max\;}+\mu }$$

Considering the fluctuation of auto-correlation, the discrete trust value is as follows:

(5) $${DIS}_{i}(t)=\left\{1-\begin{array}{ll} 0 & {\;\;\;\;\;\;\;\;d}_{i}(t)>d_{min}\\ \frac{{\;d}_{i}(t)+\beta }{d_{max}+\beta } & \varepsilon <{\;d}_{i}(t)\leq d_{min}\\ 1 & {\;\;\;\;\;\;\;\;\;\;\;\;\;d}_{i}(t)\leq \;\varepsilon \end{array}\right.$$

where ${\;d}_{i}(t)$ is the difference between the current time and the auto-correlation value of the previous time, $d_{max}$ and $d_{min}$ are the maximum and minimum differences at the current time, respectively, β is the empirical value, ε is the absolute difference.

In addition, in order to improve the sensitivity of the model and accelerate the response to malicious processes, we introduced a reward and punishment function, which will accelerate the decline of the trust value when malicious programs invade, allowing for faster detection of malicious processes. Set *P* (0) = 1 at moment *t* = 0, and the reward and punishment function P(t) is expressed as follows:

(6) $$P(t+1)=P(t)\cdot {log}_{2}\left(1+\frac{T_{i}^{\;dir\;}(t)+\delta }{T_{i}^{\;avg\;}(t)+\delta }\right)$$

where $T_{i}^{\;avg\;}(t)$ is the average value of the direct trust value, expressed as:

(7) $$T_{i}^{avg}(t)=(\sum_{t=0}\;T_{i}^{dir\;}(t))/t$$

The above reward and punishment function introduces a logarithmic function, which aims to restrain the growth of malicious process trust values effectively and stabilize the trust values of normal processes. The direct trust value in the above formula can be expressed as:

(8) $$T_{i}^{dir}(t)=W_{i}(t)\cdot {DIS}_{i}(t)\cdot P(t)$$

The direct trust value expression above is composed of three parts, namely, the work trust value, the discrete trust value, and the reward and punishment function. We set the initial value of the reward and punishment function P(t) = 1, in order to continue iteration.

### 3.3. Reciprocal Trust Value Calculation

The performance parameters between processes will be affected by other related processes, and their auto-correlation values will also fluctuate. When malicious processes invade, the auto-correlation values and trust values of their related processes will also fluctuate, which will interfere with system detection. Therefore, we introduced the concept of classification, which classifies processes according to direct trust values and auto-correlation values through k-means cluster analysis. After data processing, it is found that k-means cluster analysis has a faster clustering speed and a higher accuracy of clustering results, which is more suitable for the proposed trust evaluation model. When malicious processes are in one cluster, several processes in the cluster may be affected by malicious processes. Therefore, through a continuous iteration of the classification, the reciprocal trust values of the processes are calculated to further identify the malicious processes. The specific calculation procedure is as follows: suppose that the processes are divided into *n* cluster groups, then the cluster set can be expressed as:

*G* = [*G*_1_, *G*_2_, ……*G*_n_] (9)

Then, the set of the *j*-th cluster group is:

*G_j_* = [ (*T*_1_, *C*_1_), (*T*_2_, *C*_2_), ……, (*T_M_*, *C_M_*), …… (*T_N_*, *C_N_*)] (10)

where *T* is the direct trust value, *C* is the auto-correlation value, let the cluster center of the *m*-th cluster group be (*T_M_*, *C_M_*), then the Euclidean distance from the *i*-th point in the cluster to the cluster center is Di, denoted as:

(11) $$D_{i}=\sqrt{{\left(T_{i}-T_{M}\right)}^{2}+{\left(C_{i}-C_{M}\right)}^{2}}$$

In order to determine the degree of dispersion of points in a cluster group, the discrete weight *w*(i) is defined as the distance from the point to the center of the cluster divided by the maximum distance from all points in the group to the center of the cluster. The specific formula is as follows:

(12) $$w(i)=\frac{Di}{max(D1,D2,D3\ldots \ldots \ldots \ldots \ldots .DN)}$$

Based on the above formula, the reciprocal trust value is obtained, which is expressed as follows:

(13) $$T_{i}^{rec}(t)=(1-\gamma \cdot w\left(i\right))\cdot T_{n}^{direct}(t)$$

where γ is the adjustment coefficient, and its magnitude is related to the rate of change in the reciprocal trust value.

### 3.4. Integrated Trust Value Calculation

To better adapt to changes in the processes, adaptive weights are introduced in this section. In previous studies, the weights assigned to trust values depend on subjective judgment, which is not flexible enough to reflect process changes in the system. To avoid subjective factors and make the results more objective, this module uses the optimal entropy weight method to evaluate the process parameters. Firstly, according to the k-means clustering analysis results, the processes are analyzed in a fine-grained manner, and processes are roughly divided into three categories: trusted processes, suspected trusted processes, and suspected malicious processes. Different direct trust weights are assigned to the three categories of processes. The formula of direct trust weight is as follows:

(14) $$w_{i}^{dir}=\left\{\;\begin{array}{ll} \frac{1}{2}\;(1-W_{i}) & \;\;Suspected\;malicious\;\\ \frac{1}{2}\;(1+W_{i}) & \;\;\;\;\;Suspected\;trusted\;\\ \;1 & \;\;\;\;\;\;\;\;\;\;\;\;\;\;\;\;\;\;\;\;\;Trusted\; \end{array}\;\right.$$

where $w_{i}^{dir}$ is the preprocessing weight of the *i*-th process, and $W_{i}$ is calculated by the optimal entropy weight method, the calculation process is as follows.

First, the mean values of the parameters of the processes during the booting and fluctuation states are extracted and recorded in the form of a matrix as follows:

(15) $$F_{martix}=\left[\begin{array}{cccc} \bar{\alpha_{1}} & \bar{\beta_{1}} & \bar{\gamma_{1}} & \\ \vdots & \vdots & \vdots & \\ \bar{\alpha_{n}} & \bar{\beta_{n}} & \bar{\gamma_{n}} & \end{array}\right]$$

where $\bar{\alpha }$, $\bar{\beta }$ and $\bar{\gamma }$ are, respectively, the average values of CPU usage, memory usage, and I/O, n represents the number of processes.

Then, since the weight of each parameter in the matrix should be defined objectively, the entropy weight method is used to calculate the weight of each parameter. In order to optimize the calculation results, we add constraint conditions to the basis of the entropy weight method to find the optimal solution. First, define $p_{ij}$ as the preliminary evaluation result of each parameter in ${\;F}_{martix}$; the entropy weight method is defined as follows:

(16) $$p_{ij}=\frac{1-H_{ij}}{n-\sum_{j=1}^{n}\;H_{ij}+H_{min}}$$

where $H_{ij}$ is the information entropy of each parameter in the matrix, if the proportion of each parameter in this type of parameter is expressed as ${\;h}_{ij}$, then $H_{ij}$ can be written:

(17) $$H_{ij}=-\;\sum_{i=1}^{n}{\;h}_{ij}ln\left(h_{ij}\right)$$

Then, ${\;e}_{ij}$ represents the evaluation weight of each parameter evaluation result, and the constrain of ${\;e}_{ij}$ as follows:

(18) $$\sum_{i=1}^{n}\;{\;e}_{ij}\;=1,\;0\;{<e}_{ij}<1$$

In addition, in order to optimize the information entropy of evaluation results, the solution equation is defined as:

(19) $$min\left\{{-\sum }_{j=1}^{m}\;\sum_{i=1}^{n}\;{\;e}_{ij}ln\left(e_{ij}\right)\right\}$$

where *m* is the number of parameter types, the expression of the optimal solution can be preliminarily expressed as:

(20) $$min\left\{{-\sum }_{j=1}^{m}\;\sum_{i=1}^{n}\;{\;e}_{ij}ln\left(e_{ij}\right)\right\}\;s.t:\;\;{\;\sum }_{i=1}^{n}\;{\;e}_{ij}=1\;0\;{<e}_{ij}<1$$

In order to reduce the expected deviation generated by evaluation result $p_{ij}$ obtained by entropy weight method, evaluation deviation *ED* is defined as:

(21) $$ED=-\frac{1}{n}\sum_{j=1}^{m}\sum_{i=1}^{n}e_{ij}\left(\sum_{i=1}^{n}\;{\left[\left(p_{ij}-{\bar{p}}_{j}\right)\right]}^{2}\right)$$

Finally, the expected deviation and the information entropy are combined into an optimal solution equation as follows:

(22) $$min\left\{-\sum_{j=1}^{m}\sum_{i=1}^{n}e_{ij}ln\left(e_{ij}\right)+\frac{1}{n}\sum_{j=1}^{m}\sum_{i=1}^{n}e_{ij}\left(\sum_{i=1}^{n}{\left[\left(p_{ij}-{\bar{p}}_{j}\right)\right]}^{2}\right)\right\}\;s.t:\;\;{\;\;\sum }_{i=1}^{n}\;e_{ij}=1\;\;\;\;\;\;\;\;\;\;\;\;\;\;\;\;0<e_{ij}<1$$

Then, the optimal solution formula is solved using the Lagrange multiplier method to obtain $e_{ij}$ as follows:

(23) $$e_{ij}=\frac{e^{-\sum_{i=1}^{n}{\left(p_{ij}-{\bar{p}}_{j}\right)}^{2}}}{\sum_{j=1}^{m}e^{-\sum_{i=1}^{n}{\left(p_{ij}-{\bar{p}}_{j}\right)}^{2}}}$$

So, from the above result, $W_{i}$ is expressed as:

(24) $$W_{i}=\frac{\sum_{j=1}^{m}e_{ij}}{\sum_{j=1}^{m}\sum_{i=1}^{n}e_{ij}}$$

Similarly to the direct trust weights, the reciprocal trust weights are also analyzed in a fine-grained manner:

(25) $$\;w_{i}^{rec}=\left\{\begin{array}{ll} \frac{1}{2}\;(1-\sum_{j=1}^{m}\left(\frac{\omega_{j}^{dir}}{\sum_{k=1}^{N}\omega_{k}^{dir}}\right))\cdot \omega_{i}^{dir} & \;Suspected\;malicious\;\\ \frac{1}{2}\;(1+\sum_{j=1}^{m}\left(\frac{\omega_{j}^{dir}}{\sum_{k=1}^{N}\omega_{k}^{dir}}\right))\cdot \omega_{i}^{dir} & \;\;\;\;Suspected\;trusted\;\\ \;\;\;1 & \;\;\;\;\;\;\;\;\;\;\;\;\;\;\;\;\;\;\;Trusted\; \end{array}\right.$$

where *N* is the number of processes in the cluster, and *m* is the number of the same processes in the cluster at the previous and current time. Generally speaking, the smaller the change in the number of clusters, the more stable the cluster, so the process is relatively trusted.

The integrated trust value is weighted by the direct trust value and the reciprocal trust value. The specific calculation method is as follows:

(26) $$T_{i}^{Int}(t)={a\cdot T}_{i}^{dir}(t)+b\cdot T_{i}^{rec}(t)$$

In the formula, a and b are defined as follows:

(27) $$a=\frac{\omega_{i}^{dir}(t)}{\omega_{i}^{dir}(t)+\omega_{i}^{rec}(t)}$$

(28) $$b=\frac{\omega_{i}^{rec}(t)}{\omega_{i}^{dir}(t)+\omega_{i}^{rec}(t)}$$

In this section, four modules of the PB-ATEM trust evaluation model are introduced in detail. In the signature calculation module, process performance parameters are used to obtain unique process identification, and the auto-correlation values are calculated according to the context. In the direct trust module, the direct trust values are calculated using working trust values, discrete trust values, and the reward and punishment function. In the reciprocal trust module, the processes are classified using the cluster analysis of k-means, and the reciprocal trust values are calculated according to the discrete degree and direct trust values of processes in the cluster. In the integrated trust module, the k-means and optimal entropy weight methods are used to perform a fine-grained analysis of processes, and different adaptive weights are given to each process based on its type. The integrated trust values for the processes are obtained by the direct trust values and reciprocal trust values based on adaptive weights.

## 4. Experimental Results and Analysis

### 4.1. Simulated Experimental Environment and Results

In this paper, the VMWARE virtual machine is used for experiments to simulate the process operation of the OT sensor terminal, and the Ubuntu 22.04 operating system is selected. A total of 80 processes were selected in the sensing terminal. In order to simulate the real world, these processes include device management processes, data collection processes, data processing processes, and communication processes, covering the key processes of IoT terminal sensors. These processes work together and are responsible for data acquisition, real-time monitoring, automatic control, and other functions. About 15% of the processes are simulated as malicious processes. We set up a series of trigger scripts inside the specified processes to make those processes perform malicious behavior as required. Furthermore, we divided the types of malicious behaviors into three categories. The simulation mechanisms are detailed in Table 1. The malicious process simultaneously performs all three attacks described in the table.

To verify that the model can not only respond quickly to malicious processes but also ensure the stability and precision of the trust evaluation, we selected the trust evaluation model in the literature [12] and the BLTM trust evaluation model in the literature [20], which have been widely used in the research of trust models in recent years. Both comparison models detect malicious behavior based on trust values, following a fundamental process of deriving a comprehensive trust value by calculating sub-trust values and trust weights. However, these comparison models lack adaptability, and their trust weights cannot dynamically adjust according to the detection target. Therefore, we proposed PB-ATEM to address these shortcomings and used PB-ATEM to compare with these two models.

First, in PB-ATEM, we divided the processes into three types according to the k-means cluster analysis, namely, trusted process, suspected trusted process, and suspected malicious process. Therefore, the value of k in k-means is fixed to three, which means that all processes are divided into three groups, and the characteristics of each type of process are described in Table 2.

**Figure 3:**
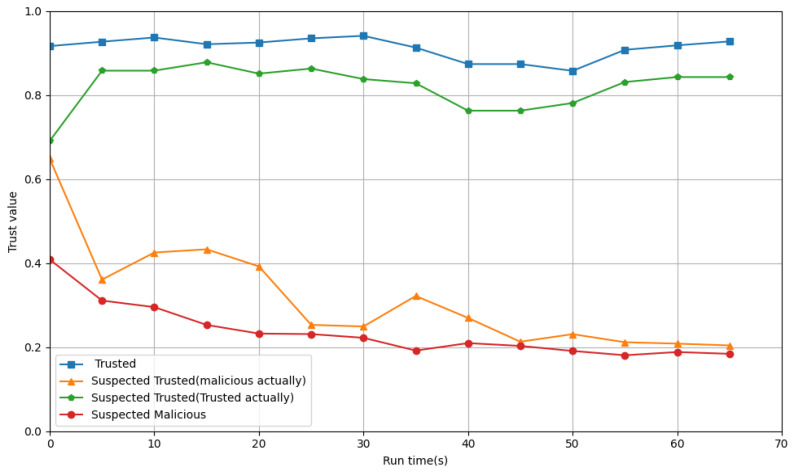
Changes in trust values for different categories of processes.

Figure 3 shows how the integrated trust values of these four types of processes change over time in PB-ATEM. As shown in Figure 3, since each type of process is assigned different adaptive weights, they have different initial trust values. Processes classified into trusted categories remained at a stable level throughout the monitoring period. For suspected trusted processes, we artificially modified the start parameters in the scripts of some trusted processes, so that the parameters of these processes will fluctuate slightly. Then, these trusted processes and some malicious processes are classified as suspected trusted processes. When processes enter the stable phase, the trust values of these trusted processes will soon return to the normal level. For malicious processes initially classified as suspected trusted by PB-ATEM, we retrieved their runtime parameters and found that this type of malicious process initially disguises itself as a normal process by faking low power consumption and low CPU usage, simulating a memory leakage pattern. However, when the malicious process launches an attack, its performance parameters exhibit significant fluctuations over a period of time. PB-ATEM detects these fluctuations and applies corresponding penalties while adaptively tracking the malicious process until it is fully identified. For suspected malicious processes, their trust values will remain low and continue to decline.

**Figure 4:**
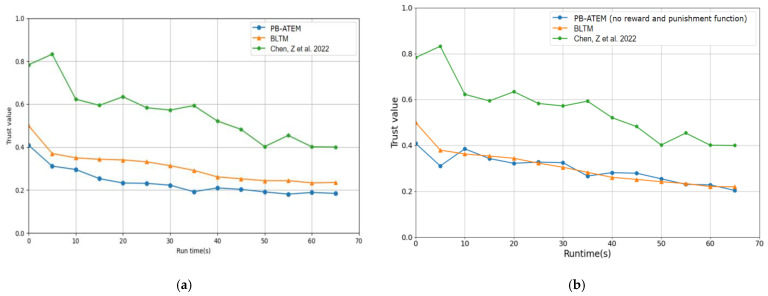
(**a**) PB-ATEM (with reward and punishment functions) vs. the Comparison models; (**b**) PB-ATEM (without reward and punishment functions) vs. the Comparison models. The data with green color from [12].

In Figure 4, the changes in integrated trust values of malicious processes in the three models are compared over time. In Figure 4a, it can be seen that in the acceleration of the reward and punishment function, the trust values of the PB-ATEM for malicious processes have been declining over time. In contrast, the models in BLTM and the model in the literature [12] have not established effective reward and punishment functions. Although these models show obvious responses to malicious processes, the trust values for malicious processes in the two models do not change significantly over time, and sometimes malicious processes may not be identified. To intuitively show the role of the reward and punishment function in PB-ATEM, we removed the function from PB-ATEM and compared it with the other two models. As shown in Figure 4b, it is found that the response of PB-ATEM without the reward and punishment function to malicious behavior is slower than before, almost the same as BLTM, because our reward and punishment function is based on the log function, and the value of function rises slowly and falls rapidly. When malicious behavior occurs, the severity of punishment will continue to increase, leading to an accelerated decline in trust value.

**Figure 5:**
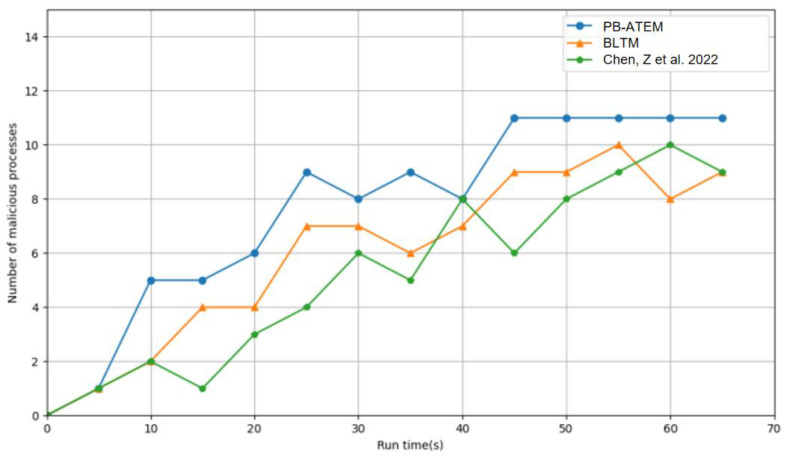
Response speed of the three models to malicious processes. The data with green color from [12].

In Figure 5, the response speed of the three models to malicious processes is compared. Due to the acceleration effect of the reward and punishment function and the filtering of the k-means cluster analysis, the PB-ATEM has a faster response speed to malicious processes, and after the model stabilizes, it is no longer affected by the interference of malicious processes. However, in the other two models, once the trust weights are artificially set, they cannot be effectively handled in the face of some special situations (such as non-malicious fluctuation), so the two models are always in an unstable state, and the response speed is not satisfactory.

**Figure 6:**
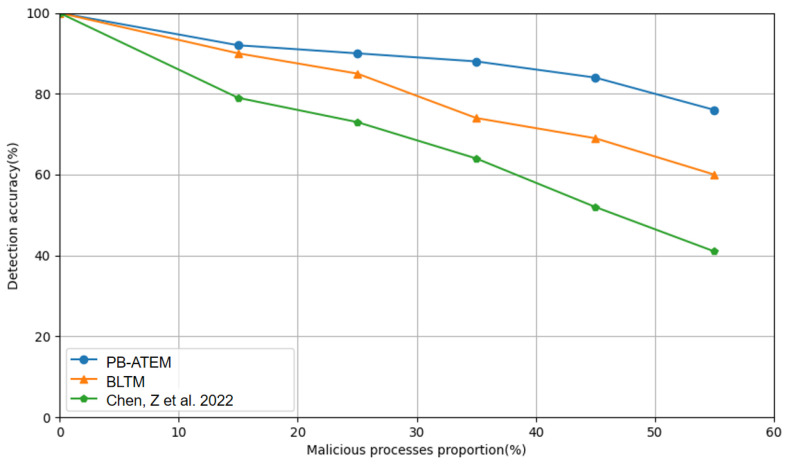
The accuracy of the three models in detecting malicious processes in different proportions of malicious processes. The data with green color from [12].

Figure 6 compares the accuracy of the three models in detecting malicious processes with different proportions of malicious processes. In this paper, the column of malicious processes ratio is set between 0 and 55%. As the proportion of malicious processes increases, the accuracy of the PB-ATEM can be maintained at a higher level, with an average of more than 75%. Even if the proportion of malicious processes increases, k-means cluster analysis can still distinguish them well by calculating the changes in auto-correlation values and direct trust values, along with the accelerated response of the reward and punishment function to malicious processes. However, the model in the literature [12] and the BLTM model do not perform classification and acceleration operations, which results in normal processes being vulnerable to the influence of malicious processes. This leads to misjudgment of the trust evaluation model and reduced precision.

### 4.2. Contrast Experimental Environment and Results

In this section, to minimize biases introduced by our dataset generated in a simulated environment and to ensure a more accurate comparison between PB-ATEM and the two comparison models, we utilized the dataset generated within the BLTM model environment as the benchmark dataset. The parameters in the dataset were signed using BLS before being input into the three models to enhance compatibility with their respective frameworks. We set the total number of detection targets to 60, the proportion of malicious behavior in the three models to 20%, and normal behavior to 80%, leaving the other parameters unchanged. Table 3 presents a comparative analysis of the three models in terms of resource occupancy, response speed, and detection accuracy under the same dataset.

As shown in Table 3, in this experiment, we ran all three models simultaneously until the detection results converged. By examining the system logs, we calculated the convergence response time and average CPU usage of the models and determined the detection accuracy by calculating the proportion of successfully detected malicious behaviors. In terms of resource consumption, BLTM has the lowest computational complexity, while the model the model in the literature [12] and PB-ATEM have slightly higher complexity. In terms of response speed and accuracy, both comparison models perform worse than PB-ATEM. The fundamental reason is that neither BLTM nor the model the model in the literature [12] includes an adaptive trust weight calculation process, and their trust weights are assigned subjectively. Moreover, they lack a reward and punishment mechanism for malicious behavior. Although this reduces computational complexity to some extent, it prevents the models from better adapting to changes in target behavior, ultimately leading to deficiencies in response speed and accuracy.

### 4.3. Experimental Summary

In this section, two widely used trust evaluation models are selected and compared with the PB-ATEM. The sensitivity, response time, and detection accuracy of the models are compared. In general, PB-ATEM is adaptable to different environments. Due to the optimal entropy weight method, PB-ATEM can also be more fine-grained for different types of processes. Due to the reward and punishment function, this model can make faster adjustments in more different situations, which are not available in the other two comparison models. Therefore, PB-ATEM is more suitable for sensing terminal process detection because of its high accuracy, faster response, and smaller resource consumption

## 5. Conclusions and Future Works

This paper proposed an adaptive trust evaluation model based on the entropy weight method for sensing the terminal process. The model can calculate the unique identification of processes in the sensing terminal based on the performance parameters and the auto-correlation values according to the unique identification, allowing for the dynamic detection of changes in process behavior. At the same time, the reward and punishment functions are introduced, which are combined with k-means cluster analysis, to identify the malicious processes faster. Compared with BLTM and the model in the literature [12], this model has a higher detection rate and response speed for malicious processes, and also higher stability. Therefore, the model can reflect the changes in processes more accurately and quickly and has better performance in resisting the intrusion of malicious processes.

However, in the reciprocal trust module, the k-means clustering analysis we used may be overfitting when there are a large number of processes; however, no such situation occurred in our experiment. The 80 processes selected in our experiment are basically applicable to most sensor devices. In the future, we will further test the stability of the model under different process counts in order to work on a more effective detection of sensor terminal threats.

## Figures and Tables

**Table 1:** Malicious Mechanism.

Malicious Behavior	Mechanism
Malicious memory leak	When a process executes a malicious script, the allocated memory cannot be released correctly, resulting in a waste of system resources and a degradation of process performance.
Distributed denial of service	The malicious process launches denial-of-service (DoS) attacks on specific targets, consuming the target system resources or network bandwidth and ultimately causing it to fail in providing services.
Challenge collapsar	The malicious process uses a proxy server to hide the real identity, simulate normal interaction behavior, and send requests continuously. The goal is to exhaust the system resources and prevent it from processing normal requests.

**Table 2:** Characteristics Description.

Type	Characteristic
Trusted process	Normal operation and stable parameter change
Suspected trusted process (Trusted)	Process with slightly fluctuating parameters but no malicious behavior
Suspected trusted process (Malicious)	Process with slightly fluctuating parameters but malicious behavior, such as malicious shell scripts
Suspected malicious process	Processes that are highly likely to engage in malicious behavior, such as DDoS and modification of sensitive files

**Table 3:** Performance comparison.

	Average CPU (%)	Response Speed (s)	Accuracy Rate (%)
PB-ATEM	19.26	43.847	93.5
BLTM	16.97	68.946	87.1
Chen, Z et al. 2022 [12]	17.35	78.125	76.8

## Data Availability

No new data are generated in this article.
